# Metastatic Adenocarcinoma of the Prostate Masquerading as a Splenic Flexure Colonic Polyp: A Diagnostic Conundrum

**DOI:** 10.7759/cureus.64959

**Published:** 2024-07-19

**Authors:** Zakaria W Shkoukani, Alaa Chamsin, Mohamed I Abdulmajed

**Affiliations:** 1 Department of Urology, St Helens and Knowsley Teaching Hospitals NHS Trust, Liverpool, GBR

**Keywords:** splenic flexure, polyp, colonic metastasis, colonoscopy, prostate cancer

## Abstract

Metastasis of prostate cancer to the gastrointestinal tract is infrequently described in the literature, with only a limited number of cases reporting metastasis to the rectum. We present a rare case of a 74-year-old man initially presenting with symptomatic anemia and weight loss. A colonoscopy revealed a sessile polyp in the splenic flexure. Microscopic examination of the specimen showed micro-acinar structures, and immunohistochemical staining was positive for prostate-specific antigen (PSA) and NKX3.1. Subsequently, elevated serum PSA levels and staging computed tomography (CT) findings, in conjunction with histopathological analysis, confirmed a diagnosis of metastatic prostate adenocarcinoma.

## Introduction

Prostate cancer represents a significant global health burden, ranking as the second most prevalent malignancy amongst men worldwide [1]. Data from the Global Cancer Observatory (GCO) platform indicate that in 2022, there were 1,466,680 new diagnoses of prostate cancer, accounting for 7.3% of all new cancer cases and resulting in 396,792 deaths. Consequently, prostate cancer is recognised as the second leading cause of cancer-related mortality in men, following lung cancer [2]. Identified risk factors include age, ethnicity, obesity, and a positive family history [1].

The prognosis for metastatic prostate cancer remains poor, with five-year survival rates reaching 30.5% [2]. The most common metastatic sites include local and distant lymph nodes, the axial skeleton (and bone in general), the lungs, the liver, and, less frequently, the brain [1]. Metastasis to the colon is fairly rare; a large retrospective study reported that only approximately 2.7% of prostate cancer patients exhibited metastases to the gastrointestinal tract, excluding the liver [3]. Herein, we report a rare case of metastatic prostate cancer, incidentally diagnosed from a splenic flexure sessile polyp biopsy during colonoscopy.

## Case presentation

A 74-year-old Caucasian male, a retired factory worker, initially presented to his local general practice with complaints of unintentional weight loss and generalised fatigue. Until recently, his Eastern Cooperative Oncology Group (ECOG) performance status was 0, and his medical history was significant only for vitamin B12 and folic acid deficiency [4]. He had ceased smoking over 30 years ago and had a body mass index (BMI) of 24. Notably, he had no family history of colonic or prostate cancer.

General physical examination was unremarkable. Blood investigations revealed anaemia with a haemoglobin level of 83g/L and normal kidney function, evidenced by an estimated glomerular filtration rate (eGFR) of over 90 mL/min/1.73m^2^. A blood film showed a leukoerythroblastic picture with occasional teardrop red blood cells, metamyelocytes, and promyelocytes with polychromatic cells, suggestive of possible myelofibrosis. A myeloma screen was negative, prompting a referral for a haematological evaluation.

**Figure 1:**
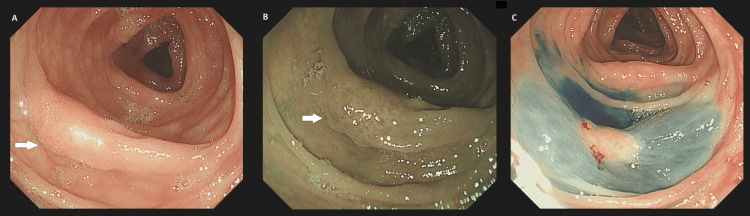
Sessile polyp (indicated by white arrows) detected at the splenic flexure on colonoscopy. A: Colonic lesion on the mucosal fold seen under normal white light. B: Colonic lesion seen under narrowband imaging. C: Colonic lesion after an attempt to lift away from the submucosa using an EMR solution. Difficulty in lifting the lesion away from the submucosa is a concerning feature for neoplasia. EMR: endoscopic mucosal resection

Faecal immunochemical testing (FIT) was positive at 49.4 µg/g, leading to the arrangement of gastrointestinal endoscopy to exclude malignancy. Upper gastrointestinal endoscopy (oesophagogastroduodenoscopy (OGD)) reported normal findings. However, a colonoscopy revealed a small, flat, sessile polyp at the splenic flexure, as demonstrated in Figure 1. Endoscopic mucosal resection (EMR) was deemed difficult and therefore biopsies were taken and a staging computed tomography (CT) scan was organised.

**Figure 2:**
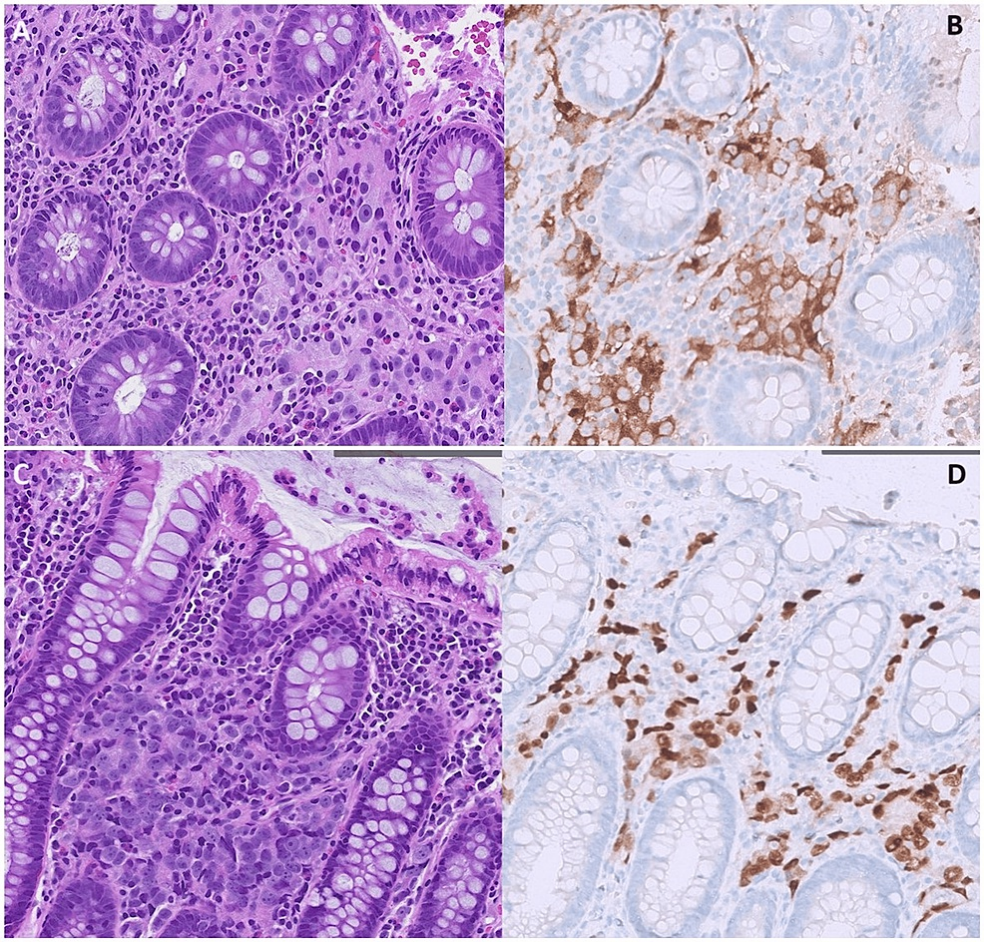
Histological and immunohistochemical findings confirming a diagnosis of metastatic prostate disease. A: Haematoxylin and eosin staining: pale neoplastic cells with prominent nucleoli interspersed between colonic crypts. B: PSA immunohistochemistry: brown cytoplasmic staining highlights neoplastic cells. C: Haematoxylin and eosin staining: sheets of neoplastic cells in the lamina propria of the colonic mucosa. D: NKX3.1 immunohistochemistry: brown nuclear staining highlights neoplastic cells. PSA: prostate-specific antigen

Histopathological analysis of the colonic biopsy revealed fragments of large intestinal mucosa, with the lamina propria and submucosa containing sheets and nests of neoplastic cells occasionally forming acinar structures. Immunohistochemical analysis of the neoplastic cells showed diffusely strong positive staining for prostate-specific antigen (PSA) and homeobox protein NKX3.1. There was also focal positive staining for CK20 and caudal type homeobox 2 (CDX2), which was deemed insignificant. Other markers, such as CK7, TTF-1, and GATA-3, were negative, while CD56 and synaptophysin showed patchy positivity. In conclusion, histological findings, as displayed in Figure 2, were suggestive of a poorly differentiated adenocarcinoma with an immunohistochemical profile favouring a prostatic primary. 

**Figure 3:**
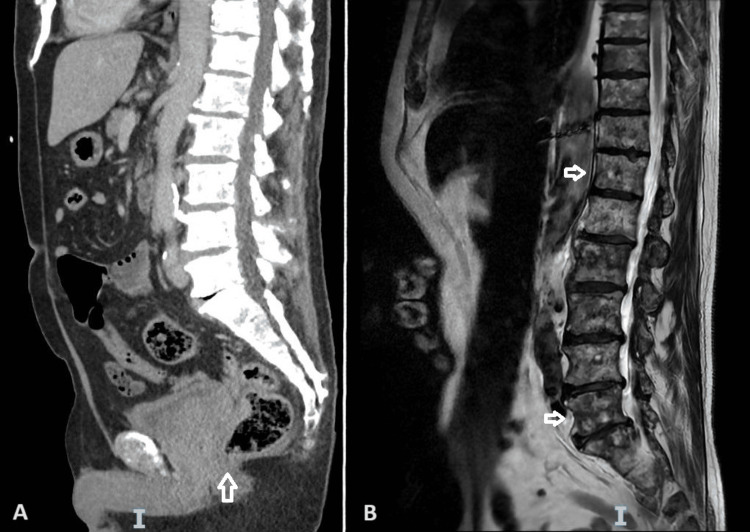
Sagittal views of staging CT scan of the abdomen and pelvis, as well as MRI of the lumbosacral spine. A: Sagittal view of staging CT scan (chest, abdomen, and pelvis) clearly showing irregular appearances of the prostate gland with loss of adjacent fat planes and tumour extension into the seminal vesicle and lower rectum (as indicated by white arrow). B: Sagittal view of MRI lumbosacral spine showing diffuse skeletal metastases with multiple lytic and sclerotic lesions (as indicated by white arrows) but no evidence of cord compression. CT: computed tomography; MRI: magnetic resonance imaging

Subsequently, CT imaging of the chest, abdomen, and pelvis (as seen in Figure 3) confirmed an irregular outline of the prostate gland with loss of adjacent fat planes, indicating tumour extension into the seminal vesicles and invasion of the lower rectum. Bilateral low-volume iliac chain and para-aortic lymph nodes were also noted. Additionally, extensive mixed lytic and sclerotic metastases were present throughout the bones (evident on sagittal magnetic resonance imaging (MRI) in Figure 3B). The overall radiological appearance was consistent with a locally advanced prostate tumour causing abdominal and pelvic lymphadenopathy, as well as extensive bone metastases, corresponding to a radiological staging of T4 N1 M1c. Serum PSA levels were markedly elevated at 5915 µg/L (normal PSA value adjusted for patient's age is <4.5 µg/L).

Histopathological analysis of limited trans-perineal prostate biopsies, performed under local anaesthesia, yielded scanty tissue foci. These foci showed absent basal cell markers p63 and CK5/6, with overexpression of racemase. The pathologist concluded that these findings supported the diagnosis of poorly differentiated high-grade adenocarcinoma of the prostate.

Following the administration of androgen deprivation therapy (ADT) with lifelong triptorelin intramuscular injections (gonadorelin analogue, 22.5 mg administered once six-monthly) as well as novel therapy with apalutamide (second-generation nonsteroidal antiandrogen, 240 mg administered orally once daily), the patient's PSA levels dropped significantly from an initial peak value of 5915 µg/L to a low of 18.1 µg/L. The patient remains under the care of the oncology team, with disease response being monitored through serial PSA checks and regular reviews. Additionally, appropriate lifestyle advice was provided to avoid pseudo-metabolic syndrome.

## Discussion

Prostate cancer, despite its generally slow progression, significantly contributes to male cancer-related morbidity and mortality. Although recent advancements in cancer therapy have been made, the prognosis remains poor once the disease has metastasised [2]. Common sites of spread include locoregional and distant lymph nodes, bones, lungs, and the liver [3]. Metastasis to the colon is an uncommon occurrence. While a few cases in the literature have reported prostate cancer presenting as rectal metastases [5-9], there are only two other documented cases of metastatic prostate cancer first being detected in a colonic polyp, highlighting the unique nature of our findings [10,11].

Three hypothesised routes for rectal invasion by prostate cancer include direct breach of Denonvilliers' fascia, lymphatic metastasis, and iatrogenic spread along a needle biopsy tract [12,13]. In our case, none of these hypotheses adequately explain the metastasis to the splenic flexure segment of the colon. Therefore, haematogenous spread, another documented route for cancer metastases discussed in the literature, is considered likely in this instance. 

The management pathways for prostate or any other cancer metastasis to the colon versus primary colorectal cancer diverge significantly and are associated with distinct prognoses. It is therefore crucial to accurately differentiate and confirm the nature of colonic polyps before initiating management strategies. A retrospective study by Lane et al. found that three out of four patients initially presenting with bowel complaints were ultimately diagnosed with prostate cancer following colectomy procedures [11]. Obtaining an accurate histological diagnosis can prove to be challenging, particularly with smaller biopsy specimens. Integrating laboratory investigations with radiological, histopathological, and immunohistochemical findings is essential for achieving diagnostic precision in such cases.

Classical histological features of prostate malignancy often include cribriform proliferation or micro-acinar appearances characterised by nuclear enlargement and prominent nucleoli. In contrast, colorectal malignancy typically features tall columnar cells with "dirty" necrosis and a surrounding stromal reaction. Immunohistochemical markers specific to prostate adenocarcinoma include PSA, prostatic acid phosphatase (PSAP), NKX3.1, P504S, and prostein (P501S). Conversely, markers such as carcinoembryonic antigen (CEA), β-catenin, cytokeratin 20 (CK20), and CDX-2 are more commonly associated with colorectal malignancy, though with CDX2 showing at least some staining in prostatic glands (both benign and malignant), SATB2 is currently favoured as a more specific marker [14,15].

In our case, histological examination revealed micro-acinar appearances, and immunohistochemical staining showed strong positivity for PSA and NKX3.1 on a splenic flexure polyp biopsy. These findings enabled our team to confirm a diagnosis of metastatic prostate adenocarcinoma promptly, initiate the appropriate treatment regimen, and avoid advocating or performing incorrect surgeries, such as colectomy as mentioned in the literature.

## Conclusions

Our findings underscore the importance of considering prostate cancer in poorly differentiated carcinoma detected on colorectal biopsies, emphasising the pivotal role of histological morphology and immunohistochemical findings in establishing an accurate diagnosis.

A comprehensive approach in the initial assessment of patients, including a thorough exploration of symptoms and investigations such as serum tumour markers, can facilitate the early identification of primary cancer and guide invasive tests like biopsies at an earlier stage, hence contributing to timely intervention and ultimately improving patient outcomes.
